# Supplementary material for: WordCommentsAnalyzer: A windows software tool for qualitative research
Source: F1000Res. 2018 Sep 4;7:536. Originally published 2018 May 3. [Version 2] doi: 10.12688/f1000research.14819.2 (PMC6137411; doi:10.12688/f1000research.14819.2)
Supplement: Supplementary file 3 [file f1000research-7-17614-s0002.tgz › b2a79496-4ada-47c4-9682-a99ce867836d.zip!/Eight Iranian Health Journals/Journal of Knowledge and Health_13.docx]

© Journal of Knowledge and Health. This work is licensed under CC BY 4.0 (https://creativecommons.org/licenses/by/4.0/).

Volume & Issue: دوره 10، شماره 1: 1394

Title: تأثير بتا استرادیول بر نسبتBcl2/BAX نورون‌هاي هیپوکمپ موش‌هاي صحرايي متعاقب القاي فشار روانی پس از سانحه (PTSD)

http://knh.shmu.ac.ir/index.php/site/article/view/513

مقدمه: فشار روانی پس از سانحه (PTSD) نوعی اختلال روانی است که در پی مواجه با سوانح شدید بروز می‌کند. درPTSD  حجم هیپوکامپ متناسب با شدت بیماری، کاهش پیدا می‌کند. هدف مطالعه القاي PTSD و ارزیابی شاخص‌های آپوپتوزی و ضد آپوپتوزی در هیپوکمپ و متعاقب آن بررسی آثار تزریق مکرر بتا استرادیول بر شاخص‌های مذکور می‌باشد. مواد و روش‌ها: از مدل SPS برای القاي بیماری استفاده شد. به این ترتیب که موش‌های صحرايی نر نژاد ویستار 2 ساعت در مقیدکننده نگهداری شده و سپس 20 دقیقه جهت شنای اجباری در آب قرار گرفتند. 15 دقیقه بعد با اتر بیهوش شدند. یک روز بعد حیوانات در دستگاه ترس شرطی قرار گرفته و شوک الکتریکی دریافت کردند. 7 روز بعد حیوانات بدون دریافت شوک مجدداً در دستگاه قرار گرفتند و میزان بی‌حرکتی آنها سنجیده شد. آزمون رفتاری دوم، 4 روز بعد صورت گرفت. تزریق β-استرادیول (دوز g/kgμ 45) یا روغن کنجد بلافاصله پس از شوک و در طول یک هفته روزانه یک‌بار صورت گرفت. سطوح پروتئین‌های Bcl2 و  BAXهیپوکمپ با روش ایمنوهیستوشیمی بررسی شد. نتايج: ترس شرطی شده در گروه بیمار در مقایسه با گروه کنترل به‌صورت معني‌داری تشدید یافت. تزریق زیر جلدی β-استرادیول پاسخ‌های رفتاری ناشی از القاء PTSD را به‌طور معنی‌داری کاهش داد. در گروه بیمار، نسبت Bcl-2/Bax در هیپوکامپ به‌طور معنی‌داری کاهش يافت. این اثر در گروه تیمار شده با β-استرادیول به‌طور معنی‌داری معکوس شد. نتيجه‌گيري: β-استرادیول (دوز g/kgμ 45) با افزودن نسبت Bcl-2/Bax در هیپوکامپ می‌تواند از آپوپتوز القا شده توسط مدل SPS جلوگیری کند.

Title: عوامل پیش‌بینی‌کننده كيفيت زندگي در بيماران مبتلابه صرع

http://knh.shmu.ac.ir/index.php/site/article/view/534

مقدمه: پیامدهای جسمی، روانی و اجتماعی بيماري صرع کیفیت زندگی بيماران مبتلا را به شدت تحت تأثير قرار می‏دهد. مطالعه حاضر با هدف بررسي عوامل پيش‌بيني‌كننده کيفيت زندگي بيماران مبتلا‌به صرع و شناخت عواملي با بيشترين اثر انجام شد. مواد و روش‌ها: اين مطالعه مقطعی مقايسه‌اي می‌باشد كه بر روي 100 بیمار صرعي و 100 فرد سالم همراه بيمار مراجعه‌كننده به كلينيك بيمارستان 22 بهمن شهرستان مشهد انجام شد. ابزار پژوهش پرسشنامه استاندارد كيفيت زندگی سازمان جهاني بهداشت بود. براي تعيين عوامل پیش‌بینی‌کننده كيفيت زندگي مبتلایان به صرع از مدل رگرسيون خطي چند‌گانه استفاده گرديد. نتايج: ميانگين نمره كلي کیفیت زندگي بيماران و افراد سالم تفاوت نداشت و تنها در حيطه‌هاي جسمي (001/0P<)، محيط (002/0P=) و اجتماعي (001/0P<) تفاوت آماري معنی‌دار مشاهده شد. نتايج رگرسيون خطي چندگانه نشان داد متغيرهاي سن، جنس، سطح تحصيلات، نوع صرع و تعداد حملات صرع در ماه به‌عنوان عوامل پیش‌بینی‌کننده مستقل کیفیت زندگی کلی در مبتلايان به صرع بودند. در تمام حيطه‌هاي كيفيت زندگي، افزايش تعداد حملات صرع نمره كيفيت زندگي را كاهش مي دهد و زنان نسبت‌به مردان نمره كيفيت زندگي بالاتري داشتند. نتيجه‌گيري: سن، جنس، سطح تحصيلات، نوع صرع و تعداد حملات صرع به‌عنوان عوامل مهم پيشگويي‌كننده سطح كيفيت زندگي كلي بيماران مبتلا‌به صرع تعيين شدند. بيشترين اثر مربوط‌به تعداد حملات صرع و جنسيت زن بود. بر‌اساس يافته‌هاي مطالعه تنها عاملي كه مي‌توان بر روي آن مداخله نمود، كنترل تعداد حملات صرع مي‌باشد كه به‌نظر مي‌رسد نقش مؤثري در ارتقاء كيفيت زندگي بيماران مبتلا‌به صرع داشته باشد.

Title: تأثير تمرين در آب بر بهزیستی روانشناختی زنان مبتلابه مولتيپل اسكلروزيس

http://knh.shmu.ac.ir/index.php/site/article/view/494

مقدمه: مولتيپل اسكلروزيس يا ام. اس. يك بيماري مزمن و ناتوان‌كننده‌ي سيستم عصبي است كه ميلين سيستم اعصاب مركزي (مغز و نخاع) را تخريب مي‌كند. شايع‌ترين عوارض اين بيماري خستگي، گرفتگي عضلات، لرزش، دوبيني، عدم تعادل و اختلال در راه رفتن است. هدف پژوهش حاضر بررسي اثر برنامه‌ي ورزش در آب به مدت 12 هفته بر بهبود بهزیستی روانشناختی بيماران ام. اس. مي‌باشد. مواد و روش‌ها: نوع تحقيق كاربردي است، لذا از میان بيماران زن مراجعه‌‌کننده به کلینیک ام. اس. بیمارستان کاشانی اصفهان، 38 نفر به‌عنوان نمونه با درجه‌ي بيماري صفر تا 5/4، ميانگين مدت بيماري 2 ± 8 سال و دامنه‌ي سني 20 تا 40 سال انتخاب و به‌صورت تصادفي به دو گروه تمرين در آب و کنترل تقسیم شدند. برنامه‌ي تمرينات برای گروه‌ آزمایش، 12 هفته، هفته‌ای سه جلسه و جلسه‌ای یک ساعت بود. بهزیستی روانشناختی بیماران قبل از تمرینات و پس از تمرینات با پرسشنامه‌ي بهزیستی روانشناختی ریف اندازه‌گیری شد. برای تحلیل داده‌ها از نرم‌افزار آماری SPSS استفاده شد. نتايج: تجزيه‌و‌تحليل داده‌های به‌دست آمده از 30 نفر از افراد مورد مطالعه، با استفاده از آمار توصیفی و تحلیل کوواریانس و میانگین‌های تعدیل شده انجام شد. یافته‌ها تفاوت معنی‌داري را در خصوص اثربخشی آب درمانی بر بهبود بهزیستی روانشناختی بیماران ام. اس. نشان داد (001/0P=). نتيجه‌گيري: اجراي تمرينات ورزشي در آب باعث بهبود بهزیستی روانشناختی بيماران ام. اس. مي‌شود.لذا باتوجه به اين نتايج به نظر مي‌رسد، متخصصان مربوطه مي‌توانند از اين گونه تمرينات به‌عنوان نوعي درمان مكمل  توام با درمان‌هاي دارويي براي كاهش عوارض باليني  بيماران ام. اس. استفاده نمايند.

Title: تأثير بخار آب و نانوسيل بر حذف آلودگي ميكروبي از دستگاه‌هاي توليد بستنی

http://knh.shmu.ac.ir/index.php/site/article/view/333

مقدمه: هدف از مطالعه، مقايسه اثر بخار آب و نانوسيل بر حذف بار ميكروبي تجهيزات كارگاه‌هاي سنتي تولید بستنی مي‌باشد. مواد و روش‌ها: در اين تحقيق ابتدا بار ميكروبي دستگاه‌هاي توليد بستني سنتي اندازه‌گيري شد، سپس هر يك از اين دو گندزدا با سطوح داخلي دستگاه توليد بستني در زمان‌هاي 5، 15 و 30 دقيقه تماس داده شد. پس از هر بار گندزدايي، در شرايط كنترل شده از سطوح مذكور نمونه‌برداري انجام و به‌روش استاندارد كشت داده شد. بر روي داده‌هاي حاصل، آزمون‌های آماري Two-Way Annova و Tukey در محيط SPSS انجام شد و در نهايت اثربخشي اين دو روش بر بار ميكروبي كل، كليفرم و اشريشياكلي مقايسه گرديد. نتايج: در اين مطالعه، بخار آب در كمترين زمان مواجهه (5 دقيقه) توانست تعداد كل باكتري‌ها، كل كليفرم و اشريشياكلي را به صفر برساند. همچنين با افزایش زمان مواجهه با نانوسيل 2 و 6 درصد، كارايي حذف كل باكتري‌ها، كل كليفرم و اشريشياكلي افزايش يافت. نتایج آنالیزهای آماری نيز نشان داد ميزان حذف كل باكتري‌ها بين زمان‌های 5 و 15 دقيقه و همچنين بين زمان‌هاي 5 و 30 دقيقه با نانوسيل 2 و 6 درصد، معنادار بود (P به‌ترتيب 01/0 و 006/0) و بين زمان‌هاي 15 و 30 دقيقه معنادار نبود (21/0P=). نتيجه‌گيري: نتايج نشان داد كه هر دو گندزدا توانايي دستيابي به استانداردهاي كيفيت ميكروبي دستگاه‌هاي توليد بستني سنتي را دارند اما بخار آب در مدت زمان كمتري از نانوسيل توانست كل باكتري‌ها، كليفرم و اشريشياكلي را به حد استاندارد برساند.

Title: طراحي، انتخاب و بهينه‌سازي توالي‌هاي تكراري كوتاه به‌منظور تشخيص بيماري‌هاي تك ژني قبل از لانه‌گزيني

http://knh.shmu.ac.ir/index.php/site/article/view/506

مقدمه: تكرارهاي پشت سر هم كوتاه يا STR (Short tandem repeats) نشانگرهای پلی‌مورفیکی هستند که در سرتاسر ژنوم پراکنده شده‌اند و امروزه کاربردهای فراوانی در ژنتیک دارند. به‌عنوان مثال این نشانگرها در تهیه نقشه‌های ژنی، ترسيم درخت فيلو ژني، پزشکی قانونی و تشخیص غیر‌مستقیم بیماری‌ها از طریق آنالیز پیوستگی مطرح هستند. یکی دیگر از کاربردهای مهم اين نشانگرها در تشخیص قبل از لانه‌گزینی یا PGD (Preimplantation genetic diagnosis) مي‌باشد. لذا باتوجه به گستردگي کاربردهای STR‌ و باتوجه به تعداد زیاد این نشانگرها در سطح ژنوم، بایستی از بین هزارانSTR  موجود، چند STR مناسب را باتوجه به نوع کاربرد انتخاب نمود. مواد و روش‌ها: در اين پژوهش بيماري آتروفي عضلاني نخاعي به‌عنوان الگويي از يك بيماري تك ژني انتخاب گرديد، سپس نشانگرهاي اطراف ژن آن جستجو گرديد، از بين صدها نشانگر، 5 نشانگر انتخاب گرديد، طيف اندازه اللي براي 5 نشانگر تعيين گرديد، سپس انطباق چند تايي انجام شد و پرايمرهاي موردنياز طراحي گرديد. در ادامه واكنش‌هاي PCR بهينه‌سازي شدند و بر روي محصولات PCR به‌دست آمده الكتروفورز مويئنه انجام شد. نتايج: به‌منظور تسریع در امر بهینه‌سازی یک مجموعه نشانگرSTR  برای کاربردهاي مختلف، در این مطالعه یک استراتژی کلی ارائه گرديد تا در کمترین زمان ممکن بتوان از وجود یک مجموعه STR مناسب استفاده نمود. به‌عنوان يك الگو، در این مطالعه کلیه مراحل طراحي و توسعه یک مجموعه نشانگر STR، برای کاربرد در تشخیص قبل از لانه‌گزینی بیماری آتروفي عضلاني نخاعي يا SMA (Spinal muscular atrophy) آورده شده است. نتيجه‌گيري: از استراتژی ارائه شده در اين پژوهش مي‌توان ظرف مدت زمان نسبتاً كوتاه و با هزينه كم، یک مجموعه نشانگر STR برای کاربردهای مشابه طراحي نمود.

Title: مقايسه‌ي نتايج آزمايشات سرولوژي و بيوپسي در تشخيص هليكوباكتر پيلوري بيماران مبتلابه سوء هاضمه

http://knh.shmu.ac.ir/index.php/site/article/view/473

مقدمه: هليكوباكترپيلوري يك عامل مهم ايجاد عفونت مزمن معده، گاستريت مزمن، اولسر پپتيك و سرطان معده در انسان است. با استفاده از شيوه‌هاي تشخيصي سريع و ارزان و درمان آلودگي با اين ارگانيسم مي‌توان از عوارض بي‌شماري جلوگيري كرد. اين مطالعه به‌منظور مقايسه دو روش سرولوژي و هيستولوژي در تشخيص هليكوباكتر پيلوري در بيماران مبتلابه ديس‌پپسي انجام شده است. مواد و روش‌ها: اين مطالعه تحليلي بر روي 395 نفر از بيماران مبتلابه ديس‌پپسي مراجعه‌كننده به بيمارستان امام حسین(ع) شاهرود انجام شد. براي تمام بيماران آندوسكوپي انجام و بيوپسي استاندارد از 6 نقطه معده و دوازدهه گرفته شده و همزمان نمونه خون نيز جهت آزمایشات سرولوژي گرفته شد. جهت تشخيص هليكو باكتر پيلوري در مطالعات هيستولوژي از رنگ‌آميزي گيمسا و در مطالعات سرولوژي از روش اليزا جهت اندازه‌گيري تيتر IgG ضد هليكوباكتر پيلوري استفاده شده و سپس داده‌ها با محاسبه حساسیت، ویژگی و ارزش اخباری مثبت و منفی مورد بررسی قرار گرفتند. نتايج: از 395 بيمار مبتلابه ديس‌پپسي، آزمون سرولوژي هليكوباكترپيلوري در 212 نفر (7/53%) و بافت‌شناسي در 316 نفر (80%) مثبت بود. مقایسه روش سرولوژی (IgG) در مقایسه با نتایج بیوپسی دارای حساسيت 9/63%، ويژگي 3/87%، ارزش اخباری مثبت 3/95% و ارزش اخباري منفي 7/37% مي‌باشد. با استفاده از منحني راك، بهترين نقطه برش 1 واحد تعيين گرديده كه در اين نقطه حساسيت آزمون 3/95% و ويژگي آن 5/78% مي‌باشد. نتيجه‌گيري: باتوجه به بالا‌بودن ویژگی آزمون سرولوژي و بالا‌بودن نسبی حساسیت در مقايسه با روش بیوپسی و همچنين سادگي، سرعت و ارزان بودن اين آزمون، توصيه مي‌گردد كه از اين آزمون جهت غربالگري با نقطه برش 1 واحد استفاده گردد.

Title: بررسی رابطه امید به کار و انگیزش تحصیلی با فرسودگی

http://knh.shmu.ac.ir/index.php/site/article/view/503

مقدمه: فرسودگی تحصیلی ناشی از خستگی ذهنی و بی‌علاقه‌گی دانشجویان نسبت‌به تحصیل است كه خود تحت تأثير عوامل مختلفي مي‌باشد. امید به کار و انگیزش تحصیلی از عوامل مؤثر بر تعدیل این متغیر در بین دانشجویان محسوب مي‌گردند. هدف از این پژوهش بررسي رابطه امید به کار و انگیزش تحصیلی با فرسودگی تحصیلی دانشجویان دانشگاه علوم پزشكي شاهرود می‌باشد. مواد و روش‌ها: روش تحقیق مورد استفاده از نوع کاربردی و از لحاظ روش از نوع تحقیقات همبستگی است كه در زمره تحقيقات غيرآزمايشي قرار مي‌گيرد. با استفاده از جدول کریستی مورگان 291 دانشجو به‌صورت تصادفی ساده انتخاب شدند. ابزار مورد استفاده سه پرسشنامه استاندارد امید به کار اسنایدر، پرسشنامه انگیزش تحصیلی والرند (AMS-HS28) و پرسشنامه فرسودگی تحصیلی برسو بود. تجزیه و تحلیل داده‌ها با استفاده از آزمون تي مستقل و رگرسيون گام به گام صورت گرفت. نتایج: بین متغیرهای امید به کار و انگیزش تحصیلی با فرسودگی تحصیلی ارتباط معناداری به‌دست آمد و مشخص شد به ازاي یک واحد افزایش در امید به کار معادل 028/0 از فرسودگی تحصیلی کاسته می‌شود و بین انگیزش تحصیلی و فرسودگی تحصیلی رابطه معکوس به‌دست آمد.نتیجه‌گیری: تعادل بین بازار کار و پذیرش دانشجویان در رشته‌های موردنیاز می‌تواند امید به کار و انگیزش تحصیلی را در دانشجویان افزایش داده و موجب عملکرد تحصیلی بهتر و کاهش فرسودگی تحصیلی در دانشجویان شود.

Title: بررسی نشانه‌های حفاظت‌کننده شأن و عوامل مرتبط با آن در بیماران مبتلابه نارسایی قلبی

http://knh.shmu.ac.ir/index.php/site/article/view/380

مقدمه: رفتارهای مختلفی که بیماران در جهت حفظ و یا ارتقاء شأن خود انجام می‌دهند، در ایجاد حس ارزشمندی در آنان حائز اهمیت می‌باشد. پژوهش حاضر با هدف تعیین نشانه‌های حفاظت‌کننده شأن و عوامل مرتبط با آن در بیماران مبتلابه نارسایی قلبی صورت گرفته است. مواد و روش‌ها: پژوهش حاضر يك مطالعه توصيفي- مقطعی بوده و جامعه مورد مطالعه آن بیماران مبتلا به نارسایی قلبی بستری در بخش قلب بيمارستان‌هاي حضرت رسول (ص)، امام حسین (ع) و شریعتی تهران در سال 1391 بودند. در اين پژوهش، نمونه‏گيري به‌صورت غيرتصادفي (مبتنی بر هدف) بوده و تعداد 130 بيمار مبتلا به نارسایی قلبی از ميان جامعه پژوهش انتخاب گرديدند. پس از اخذ رضايت کتبی از نمونه‌ها، پرسشنامه اطلاعات دموگرافيك و همچنين پرسشنامه نشانه‌های حفاظت‌کننده شأن DCRQ))، براساس گفته‌هاي بيمار تكميل گرديد. تجزیه و تحلیل آماری با استفاده از آزمون‌های آماری پیرسون، آنالیز واریانس یک طرفه و تی مستقل شد. نتایج: میانگین سنی واحدهای مورد پژوهش 9/13±62 می‌باشد و اکثریت آنان (5/51 درصد) بی‌سواد هستند، درآمد متوسط ماهانه كمتر از یک میلیون تومان در ماه (3/92 درصد) دارند. میانگین نمره نشانه‌های حفاظت‌کننده شأن در واحدهای مورد پژوهش 8/0 ± 6/3 محاسبه گردید. بیشترین میانگین نمره مربوط‌‌به بعد پذیرش و کمترین میانگین نمره مربوط ‌به بعد عملکرد نقش است و آزمون آماری آنالیز واریانس با 05/0P<، تفاوت معناداری بین میانگین نمره نشانه‌های حفاظت‌کننده شأن در دو بعد ذکر شده نشان می‌دهد. آزمون آماری پیرسون، ارتباط معنی‌دار آماری بین سن با نشانه‌های حفاظت‌کننده شأن نشان داد. این آزمون همچنین ارتباط معنی‌دار آماری بین دفعات بستری با نشانه‌های حفاظت‌کننده شأن نشان داد. نتیجه‌گیری: از آنجا که نتایج پژوهش حاضر نشان داد بیماران مبتلابه نارسایی قلبی، سطح متوسطی از نشانه‌های حفاظت‌کننده شأن را از خود نشان دادند، لذا به‌کارگیری راهکارهایی در جهت بهبود دیدگاه و عملکرد این بیماران جهت افزایش احساس ارزشمندی در آنان توصیه می‌شود.

Title: مروری بر مقالات مرتبط با پاسخگویی به‌عنوان ویژگی ابزارهای کیفیت زندگی

http://knh.shmu.ac.ir/index.php/site/article/view/377

مقدمه: پاسخگویی، مفهومی است که در میانه‌ی دهه‌ی هشتاد بین محققین زیست- پزشکی مصطلح شده و به‌عنوان یک ویژگی اساسی پرسشنامه‌های کیفیت زندگی مرتبط با سلامتی لحاظ گردید و متفاوت از پایایی و روایی است. پاسخگویی، توانایی یک ابزار برای تعیین حداقل تفاوت‌های مهم بالینی می‌باشد. اغلب نویسندگان توافق دارند پاسخگویی بر توانایی یک معیار در اندازه‌گیری تغییر تمرکز دارد، ولی تنوع گسترده‌ای در نظرات مربوط‌به طبیعت تغییر مشخص شده وجود دارد این مطالعه مروری، برای مشخص نمودن پاسخگویی با استفاده از مقالات معتبر و دسترس انجام گردید. مواد و روش‌ها: جستجوی الکترونیکی با استفاده از پایگاه‌های داده‌ای مانند Proquest، Cinahl، Medline، Sciencedirect، Scopus و Psychinfo با کلید واژه‌های پاسخگویی و کیفیت زندگی انجام شد. نتايج: تعاریف متعدد و روش‌هایی محاسبه پاسخگویی در مطالعات مختلف بررسی گردید. در مجموع تعداد 30 عدد مقاله به‌عنوان منابع مورد استفاده قرار گرفتند. پاسخگویی به دو گروه داخلی و خارجی تقسیم‌بندی شده است. توانایی یک معیار برای تغییر در طی چارچوب زمانی مشخص، ویژگی پاسخگویی داخلی نامیده می‌شود. پاسخگویی خارجی بیانگر میزانی است که تغییرات در یک معیار در طی چارچوب زمانی مشخص به تغییرات مشابه در معیار مرجع وضعیت سلامتی مرتبط است. نتيجه‌گيري: علی‌رغم این واقعیت که بعضی از نویسندگان پاسخگویی را اساسی‌ترین ویژگی برای ارزیابی یک ابزار مي‌دانند ولی متدولوژی سنجش آن متفاوت است. اگرچه روش‌های اندازه‌گيري متفاوتی برای سنجش آن وجود دارد، ولی در طراحی و هنجاریابی ابزارهای کیفیت زندگی از یکی از روش‌ها برای اندازه‌گيري آن استفاده شده است و نیاز است در بررسی این ابزارها مورد توجه قرار گیرد.

Title: ارزيابي کیفیت زندگی در افراد مبتلابه دیابت نوع دو

http://knh.shmu.ac.ir/index.php/site/article/view/360

مقدمه: دیابت یک بیماری مزمن بوده و یکی از موارد بسیار مهم در امر مراقبت از بیماران مزمن، بررسی کیفیت زندگی بیماران است. مطالعه حاضر با هدف تعيين كيفيت زندگي بيماران دیابت نوع دو و ارتباط آن با برخي از متغيرهاي دموگرافيك انجام شده است. مواد و روش‌ها: در اين مطالعه توصيفي- مقطعي 80 بيمار مراجعه‌كننده به بيمارستان امام خمینی (ره) شهر تهران با تشخيص بيماري دیابت نوع دو شركت داشتند. نمونه‌گيري به‌روش مستمر انجام و داده‌ها با استفاده از فرم اطلاعات فردي و پرسشنامه كيفيت زندگي کیفیت زندگی زندگی بیماران دیابتی، جمع‌آوري شد. تجزيه‌و‌تحليل اطلاعات به کمک آزمون‌هاي آماري تي مستقل و آناليز واريانس يك‌طرفه انجام شده است. نتايج: کمترین میانگین نمرات کیفیت زندگی مربوط‌به بعد بیماری و بیشترین میانگین مربوط‌به بعد اجتماعی بود. ارتباط معناداری بین وجود عوارض چشمی (02/0P=) و مدت زمان ابتلا به بیماری (001/0>P) با کیفیت زندگی در حیطه جسمی وجود داشت. ارتباط درآمد افراد با کیفیت زندگی مربوط‌به بخش روانی (02/0P=)، اقتصادی (001/0>P) و نمره کلی (02/0P=) معنادار بود. همچنين ارتباط آماری معناداری بین وجود عوارض پای دیابتیک با نمره کلی کیفیت زندگی مشاهده شد (04/0P=). نتيجه‌گيري: باتوجه به ارتباط بين كيفيت زندگي و برخي متغيرهاي دموگرافيك استفاده از رويكردهاي مناسب مراقبتي ارتقاء كيفيت زندگی ازجمله اجرای برنامه‌هاي آموزشی برای بيماران مبتلابه بيماري دیابت توصيه مي‌شود.
